# Physicochemical characterization, fatty acid profile, antioxidant activity and antibacterial potential of cacay oil, coconut oil and cacay butter

**DOI:** 10.1371/journal.pone.0232224

**Published:** 2020-04-28

**Authors:** Wendell Medeiros de Azevedo, Larissa Ferreira Ribeiro de Oliveira, Maristela Alves Alcântara, Angela Maria Tribuzy de Magalhães Cordeiro, Karla Suzanne Florentino da Silva Chaves Damasceno, Nathália Kelly de Araújo, Cristiane Fernandes de Assis, Francisco Caninde de Sousa Junior

**Affiliations:** 1 Pharmaceutical Sciences Graduate Program, Federal University of Rio Grande do Norte, Natal, RN, Brazil; 2 Department of Pharmacy, Federal University of Rio Grande do Norte, Natal, RN, Brazil; 3 Food Science and Technology Graduate Program, Federal University of Paraiba, João Pessoa, PB, Brazil; 4 Department of Nutrition, Federal University of Rio Grande do Norte, Natal, RN, Brazil; Institute for Biological Research, SERBIA

## Abstract

The Amazon region is rich in genetic resources such as oilseeds which have potentially important local commercial exploitation. Despite its high concentration of bioactive compounds, cacay (*Caryodendron orinocense* Karst.) oil is poorly investigated and explored. Thus, this study focuses on the physicochemical characterization (moisture, density, and saponification, iodine, and acidity values), fatty acid composition as determined by gas chromatograph mass spectrometry (GC/MS), total phenolic content (TPC), and antioxidant activity (DPPH and ABTS radical scavenging assay) of cacay oil, coconut oil and a coconut/cacay oil blend, also known as cacay butter. The antibacterial activity of cacay oil was additionally evaluated. Our study demonstrated that cacay oil presents a high amount of polyunsaturated fatty acid (PUFA) (58.3%) with an emphasis on linoleic acid and a lower acidity value (2.67 ± 0.01 cg I_2_/g) than butter and coconut oil, indicating a low concentration of free fatty acids. In contrast, cacay butter and coconut oil presented higher saturated fatty acid percentages (69.1% and 78.4%, respectively) and higher saponification values (242.78 and 252.22 mg KOH/g, respectively). The samples showed low moisture and relative density between 912 and 916 kg/m^3^. The hydrophilic fraction of cacay oil was highlighted in the quantification of TPC (326.27 ± 6.79 mg GAE/kg) and antioxidant capacity *in vitro* by DPPH radical scavenging assay (156.57 ± 2.25 μmol TE/g). Cacay oil inhibited the growth of *Bacillus cereus* (44.99 ± 7.68%), *Enterococcus faecalis* (27.76 ± 0.00%), and *Staphylococcus aureus* (11.81 ± 3.75%). At long last, this is the first study reporting the physicochemical characterization and bioactive properties of cacay butter. Coconut oil and cacay butter showed great oxidative stability potential due to higher contents of saturated fatty acids. Moreover, cacay oil presents as an alternative source of raw materials for cosmetic and biotechnology industries due to its high concentration of PUFA and for being a rich source of phenolic compounds.

## Introduction

Interest in non-conventional sources of oils and fats has increased due to increased demand for industrial use [1]. The Amazon region is privileged and rich in genetic resources such as oilseeds, which have potentially important commercial exploitation [2].

Cacay (*Caryodendron orinocense* Karst.) is a Euphorbiaceae plant which grows along the Andes base adjacent to the Amazonian lowland [3, 4]. Cacay oil is obtained by cold pressing its seeds, and possess excellent sensorial characteristics [5]. Despite its high linoleic acid concentration and bioactive compounds such as retinol and α-tocopherol, the physicochemical, total phenolic compounds, and bioactive properties of cacay are poorly investigated [6].

Virgin coconut oil is an edible oil obtained from extracting a matured kernel from the coconut (*Cocos nucifera* L.) using either mechanical or thermal processing [7]. Coconut oil is colorless with the aroma of fresh coconut, and has mainly been used by the cosmetic industry in the health supplement area [8].

The antioxidant and antibacterial properties of other oilseeds have been reported [9, 10]. Antioxidant activity contributes to investigating the oxidative stability and bioactivity of components present in vegetable lipids, which may arouse the interest of cosmetic industries since antioxidant compounds bring improvements to human health and aesthetics [11, 12]. In addition, antibacterial properties can act as topical antiseptics, making them a powerful tool against bacterial resistance [13].

Previous studies have shown potential antioxidant for cacay oil [6] and biological activities for coconut oil [14, 15]. However, to the best of our knowledge there are no studies in the literature concerning the bioactive compounds of a coconut/cacay oil blend, also known as cacay butter, which constitutes the lipid product obtained industrially from the homogenization of cacay oil with virgin coconut oil.

Thus, this is the first study that analyzes the physicochemical characteristics and antioxidant properties of cacay butter. Furthermore, the present study aims to deepen further studies on the physicochemical and bioactive properties of cacay oil and coconut oil. These results can provide relevant information about oils and fats which in turn can be used as promising alternative sources of raw materials for the cosmetic and biotechnology industries.

## Materials and methods

### Materials

Cacay (*Caryodendron orinocense* Karst.) oil and butter and coconut (*Cocos nucifera* L.) oil were kindly provided by Plantus LTDA (Nísia Floresta, Brazil). The cacay butter used in this study is a commercial product obtained by mixing 70% coconut oil and 30% cacay oil. This proportion is used to obtain a semi-solid consistency which is better-accepted by consumers (unpublished data). Samples were stored at 4°C in plastic containers. The present study was conducted under authorization from the National System for Management of Genetic Heritage and Associated Traditional Knowledge (SisGen) no. A679EA3.

### Physicochemical characterization

Moisture and volatile matter (method Ca 2d-25), saponification (method Cd 3–25), iodine (method Cd 1d-92), and acidity (method Cd 3d-63) were determined in the cacay oil, cacay butter and coconut oil using the AOCS standard [16]. Density was determined using a densimeter (Anton Paar^®^, DAM 4500, São Paulo, Brazil) and the results were expressed as kg/m^3^ at 20°C.

### Determination of the fatty acid composition

Fatty acids were initially obtained using the conventional methylation procedure previously described by Hartman and Lago [17]. The chromatographic profile was recorded, and the percentage of fatty acids was determined by a calibration curve with methyl ester standards using a GCMS-QP2010 Gas Chromatograph Mass Spectrometer (Shimadzu, Kyoto, Japan) equipped with a DB-23 Durabound column (30 m x 0.25 mm x 0.25 μm). The temperature of the injector and the detector was set at 230°C, and the column temperature at 90°C. The elution gradient in the column was from 90 to 150°C (10°C/min), 150 to 200°C (2°C/min), and 200 to 230°C (10°C/min) in a total time of 39 minutes running with a split of 100 [18]. The gas carrier used in this assay was helium.

### Preparation of hydrophilic (HF) and lipophilic (LF) fractions

**Fig 1:**
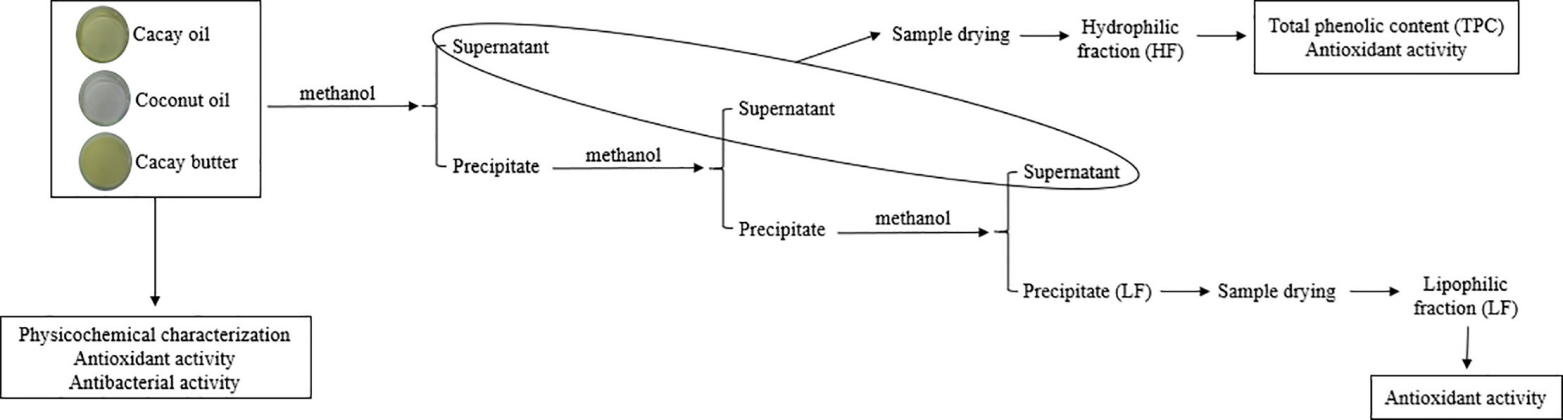
Obtaining the hydrophilic (HF) and lipophilic (LF) fractions and the performed determinations.

Fig 1 shows the methanolic extraction performed according to the methodology proposed by Arranz et al. [19], with modifications. Initially, 10 g of each sample was mixed with 20 mL of methanol at room temperature for 20 minutes. Next, the mixture was centrifuged (Centribio, 80-2B, Sao Paulo, Brazil) at 700 xg for 10 minutes at room temperature, and the supernatant was recovered. Approximately 20 mL of methanol were added to the residue, with the mixture then being shaken and centrifuged. This step was repeated twice and methanolic extracts were combined. A hydrophilic fraction (HF) (supernatant) and a lipophilic fraction (LF) (precipitate) were obtained. The fractions were dried using a rotary evaporator (Buchi, V-700, Uster, Switzerland) at 30°C and freeze-dried (LioTop, L101, São Carlos, Brazil).

### Determination of total phenolic content (TPC)

The TPC in the HF was determined according to Singleton and Rossi [20], with some modifications. Thus, 1 mL of 50% (v/v) Folin-Ciocalteau reagent and 400 μL of HF were mixed. The mixture was shaken for 3 minutes and then 400 μL of 7.5% (w/v) sodium carbonate was added. The mixture was incubated at 37°C for 30 minutes. Then the absorbance was measured at 750 nm (Biospectro, SP-220, Curitiba, Brazil). The results were expressed as milligrams of gallic acid equivalents per kilogram of sample (mg GAE/kg).

### Antioxidant activity *in vitro*

#### DPPH radical scavenging assay

DPPH (2,2-Diphenyl-1-picrylhydrazyl) radical scavenging assay in HF, LF, and total unfractionated samples (TS) was evaluated using the method by Brand-Williams et al. [21], with modifications. First, 200 μL of a DPPH ethanolic solution (0.04 mg/mL) and 40 μL of the previously diluted samples were added to each well of a 96-well microplate. A microplate reader analysis (Biochrom Asys, UVM340, Cambridge, UK) was performed at 517 nm after 25 minutes of reaction at room temperature. A calibration curve was built with concentrations from 30 to 200 μM of Trolox (6-hydroxi-2,5,7,8-tetramethylcroman-2-carboxilic acid). The results were expressed as μmol Trolox equivalent per gram of sample (μmol TE/g).

#### ABTS radical scavenging assay

ABTS (2,2′-Azino-bis (3-ethylbenzothiazoline-6-sulfonic acid)) radical scavenging assay in HF, LF and total unfractionated samples (TS) was evaluated using the method by Rufino et al. [22], with modifications. The assay was performed by adding 20 μL of sample and 280 μL of the diluted ABTS radical solution to each well of a 96-well microplate. The sample was incubated for 6 minutes and the analysis was performed in a microplate reader at 734 nm. The results were expressed in μmol Trolox equivalent per gram of sample (μmol TE/g).

### Antibacterial activity *in vitro*

Antibacterial activity in the sample with higher values of polyunsaturated fatty acids was performed according to the methodology described by the Clinical and Laboratory Standards Institute (CLSI) [23], with modifications. Six potentially pathogenic bacterial strains were tested: three Gram-negative (*Escherichia coli* ATCC 25912, *Pseudomonas aeruginosa* ATCC 27853, and *Salmonella paratyphi* ATCC 14028) and three Gram-positive (*Bacillus cereus* ATCC 11778, *Enterococcus faecalis* ATCC 29212, and *Staphylococcus aureus* ATCC 6538).

The microorganism suspension in Mueller Hinton (MH) broth (Merck, Darmstadt, Germany) was added to different sample concentrations (2.25–144.00 mg/mL) dissolved in 1% (v/v) tween 80 in a 96-well microplate. The microplates were then incubated at 35°C under shaking at 200 rpm (Quimis, Q816M20, Diadema, Brazil). The optical density at 595 nm was determined at times 0 and 24 h.

Wells containing MH broth, microorganisms, and saline solution 0.9% with 1% tween 80 were considered as positive growth control (100% bacterial growth). Vancomycin (0.4 mg/mL) was added to the Gram-positive and gentamicin (0.3 mg/mL) to the Gram-negative bacteria for the microbial sensibility profile control.

The inhibition percentage of bacterial growth was calculated according to Eq 1:

$$Inhibition\;of\;bacterial\;growth\;(\%)=\left[\frac{\Delta {Abs}_{pc}-\Delta {Abs}_{sample}}{\Delta {Abs}_{pc}}\right]x\;100$$ (1)

Where ΔAbs_pc_ represents the absorbance variation in the positive control (100% bacterial growth), and Δabs_sample_ the absorbance variation in the sample.

### Statistical analysis

Results were expressed as arithmetic mean ± standard deviation. The Statistica software (v. 8.0 from StatSoft, Inc.) program was used for *one-way* analysis of variance (ANOVA) with Tukey HSD *post hoc* test, considering a 95% confidence level (p ≤ 0.05) as a significant result.

## Results and discussion

### Fatty acid composition

The results showed that saturated fatty acids are dominant for coconut oil (78.4%) and cacay butter (69.1%), especially lauric acid (Table 1). Correia et al. [24] obtained 82.2% of saturated fatty acids present in coconut oil, with a similar amount for lauric (40.8%) and myristic (20.3%) acids. In contrast, lower saturated fatty acid content (23.6%) was found in cacay oil. Similar results were obtained by Radice et al. [6], who reported low saturated fatty acid content in cacay oil (14.3%), with a predominance of palmitic acid (10.3%).

**Table 1:** Fatty acid composition of samples.

Fatty acid	Cacay oil (%)	Coconut oil (%)	Cacay butter (%)
**Caprylic (C8:0)**	ND^a^	0.7	0.2
**Capric (C10:0)**	0.1	3.0	2.1
**Lauric (C12:0)**	ND^a^	38.4	31.8
**Myristic (C14:0)**	0.6	20.2	16.8
**Palmitic (C16:0)**	20.5	13.5	15.4
**Palmitoleic (C16:1)**	0.3	ND^a^	ND^a^
**Stearic (C18:0)**	2.3	2.5	2.7
**Elaidic (C18:1 *trans*9)**	0.7	ND^a^	ND^a^
**Oleic (C18:1 *cis*9)**	17.0	15.5	14.1
**Linoleic (C18:2 *cis*9,12)**	58.3	6.1	16.8

^a^Not detected.

Cacay oil, coconut oil, and cacay butter presented similar percentages of oleic acid among the detected monounsaturated acids. Lower results were reported by Correia et al. [24] and Pérez et al. [5] in coconut oil (9.9%) and cacay oil (11.8%), respectively. Trans-fatty acid (oleic acid isomer) was found in cacay oil (0.7%), but at a content considered insignificant when compared to Codex Alimentarius [25].

Linoleic acid (ω-6) was the most abundant fatty acid in cacay oil regarding polyunsaturated fatty acids (PUFA). Radice et al. [6] found high PUFA (85.7%) content in cacay oil, along with high linoleic acid (85.6%) content. A lower PUFA content in cacay butter can be explained due to the lower content of cacay oil, since cacay butter is a product resulting from mixing 70% coconut oil with 30% cacay oil.

Thus, the high presence of polyunsaturated fatty acids in cacay oil demonstrates its potential as a raw material for food, pharmaceutical, and cosmetic products since polyunsaturated fatty acids may have bioactive properties of industrial interest [26].

### Physicochemical characterization

Table 2 shows the results of the physicochemical characterization. Moisture content is an important parameter which influences the quality of vegetable lipids. Similar results of moisture and volatile matter were found for the samples with no statistical difference (p>0.05). Several studies report that vegetable lipids present less than 1% moisture and volatile matter [24, 27, 28]. Moreover, low moisture indicates higher resistance to microbial degradation.

**Table 2:** Physicochemical characterization of cacay oil, coconut and cacay butter.

Parameter	Cacay oil	Coconut oil	Cacay butter
Moisture and volatile matter (%)	0.93 ± 0.05^a^	0.90 ± 0.00^a^	0.99 ± 0.13^a^
Density (kg/m^3^)	915.25 ± 0.02^a^	915.86 ± 0.01^b^	912.41 ± 0.01^c^
Saponification value (mg KOH/g)	206.74 ± 1.96^a^	252.22 ± 1.85^b^	242.78 ± 1.36^c^
Iodine value (cg I_2_/g)	116.35 ± 0.67^a^	17.58 ± 0.09^b^	26.80 ± 1.17^c^
Acidity value (mg KOH/g)	2.67 ± 0.01^a^	15.71 ± 0.22^b^	13.45 ± 0.10^c^

Different lowercase letters in the same line indicate statistically significant differences (p<0.05) using One-way ANOVA, followed by the Tukey post-test.

The density of the samples evaluated in the present study agrees with Abollé et al. [29], since vegetable oils and fats have a density between 900 and 930 kg/m^3^. The saponification value found for coconut oil was similar to that obtained by Mansor et al. [30] by the cold extraction process (258.42 mg KOH/g). In contrast, cacay oil presented the lowest value among the evaluated samples, being related to the higher molecular mass of fatty acids and PUFA concentration (58.3%) [8].

The iodine value measures the degree of unsaturated fatty acids that can absorb halogens [24]. A higher iodine value was found for cacay oil, similar to that measured by Pérez et al. [5] (136.53 cg I_2_/g). This fact can be associated with the high PUFA content. On the other hand, a low iodine value was found for cacay butter due to the high saturated fatty acid concentration present (69.1%).

The acidity value of cacay oil was very similar to that found by Radice et al. [6] (2.4 mg KOH/g). In contrast, coconut oil and cacay butter showed high acidity values. It is further suggested that coconut oil and cacay butter had a high free fatty acid concentration formed by a hydrolytic rancidity process at the time of analysis. Artisanal oils are usually obtained by mechanical extraction, and are more susceptible to chemical degradation due to thermal processing and light exposure [31, 32].

### Total phenolic content

The results showed that the total phenolic content in the hydrophilic fraction of cacay oil (326.27 ± 6.79 mg GAE/kg) was higher than in the coconut oil (292.06 ± 10.04 mg GAE/kg) and cacay butter (300.45 ± 4.62 mg GAE/kg) (p = 0.007 and p = 0.013, respectively). Moreover, cacay oil has higher TPC than other oils reported in the literature. Seneviratne et al. [33] obtained a TPC for coconut oil ranging from 62.2 to 78 mg GAE/kg. Chanioti and Tzia [34] observed values of 207 and 255 mg GAE/kg for olive pomace oils obtained by Soxhlet extraction and ultrasound, respectively. It is important to highlight that the phenolic content of each vegetable oil is a function of a multiplicity of factors, such as the implemented extraction method, as well as the climatic and geographical conditions of the cultivation region [35].

### Antioxidant activity *in vitro*

The antioxidant contribution of the fractions and unfractionated samples was evaluated, as shown in Table 3.

**Table 3:** Antioxidant activity of total samples (TS), hydrophilic fractions (FH), and lipophilic fractions (FL).

Sample	Fraction	Antioxidant activity (μmol TE/g)	
		DPPH	ABTS
**Cacay oil**	HF	156.57 ± 2.25^a^	77.79 ± 0.56^a^
	LF	14.27 ± 2.15^b^	9.82 ± 0.42^b^
	TS	19.78 ± 3.18^c^	16.84 ± 1.33^c^
**Coconut oil**	HF	10.17 ± 1.20^b.d^	19.23 ± 0.98^d^
	LF	6.17 ± 0.38^d^	10.17 ± 0.73^b^
	TS	7.17 ± 0.38^d^	5.53 ± 0.98^e^
**Cacay butter**	HF	7.71 ± 1.48^d^	15.11 ± 0.72^c^
	LF	0.31 ± 0.19^e^	5.00 ± 0.45^e^
	TS	0.35 ± 0.05^e^	8.04 ± 0.63^b^

HF (hydrophilic fraction); LF (lipophilic fraction); TS (total unfractionated sample). Different lowercase letters in the same column indicate statistically significant differences (p<0.05) using One-way ANOVA followed by the Tukey post-test.

Overall, cacay oil showed a promising antioxidant capacity for the evaluated methods when compared to coconut oil and cacay butter. Radice et al. [6] showed promising results for the antioxidant capacity of cacay oil associated with high levels of α-tocopherol and linoleic acid, suggesting possible application in the cosmetic area.

HF showed significantly higher results (p<0.05) than the lipophilic fraction among the analyzed fractions for oil and cacay butter. This may be attributed to the presence of a larger TPC present in HF, since these compounds are capable of donating hydrogen to free radicals and inhibiting the propagation chain of reactions promoted by oxidative stress [36].

In contrast, no significant difference was observed between antioxidant activities in the HF and LF (p = 0.072) of coconut oil. Such results may be justified by the complex composition present in vegetable oils and the synergistic effect with other phytochemical compounds not evaluated in the present study [9, 37].

Unlike that found by Espín et al. [38], the sum of the results of HF and LF were higher than the activity expressed in samples. Similar behavior was found for faveleira (*Cnidoscolus quercifolius)* oil and its fractions. This suggests that the compounds present in the samples can lead to an antagonistic effect when mixed, thereby decreasing their antioxidant activity [27].

### Antibacterial activity *in vitro* of cacay oil

**Fig 2:**
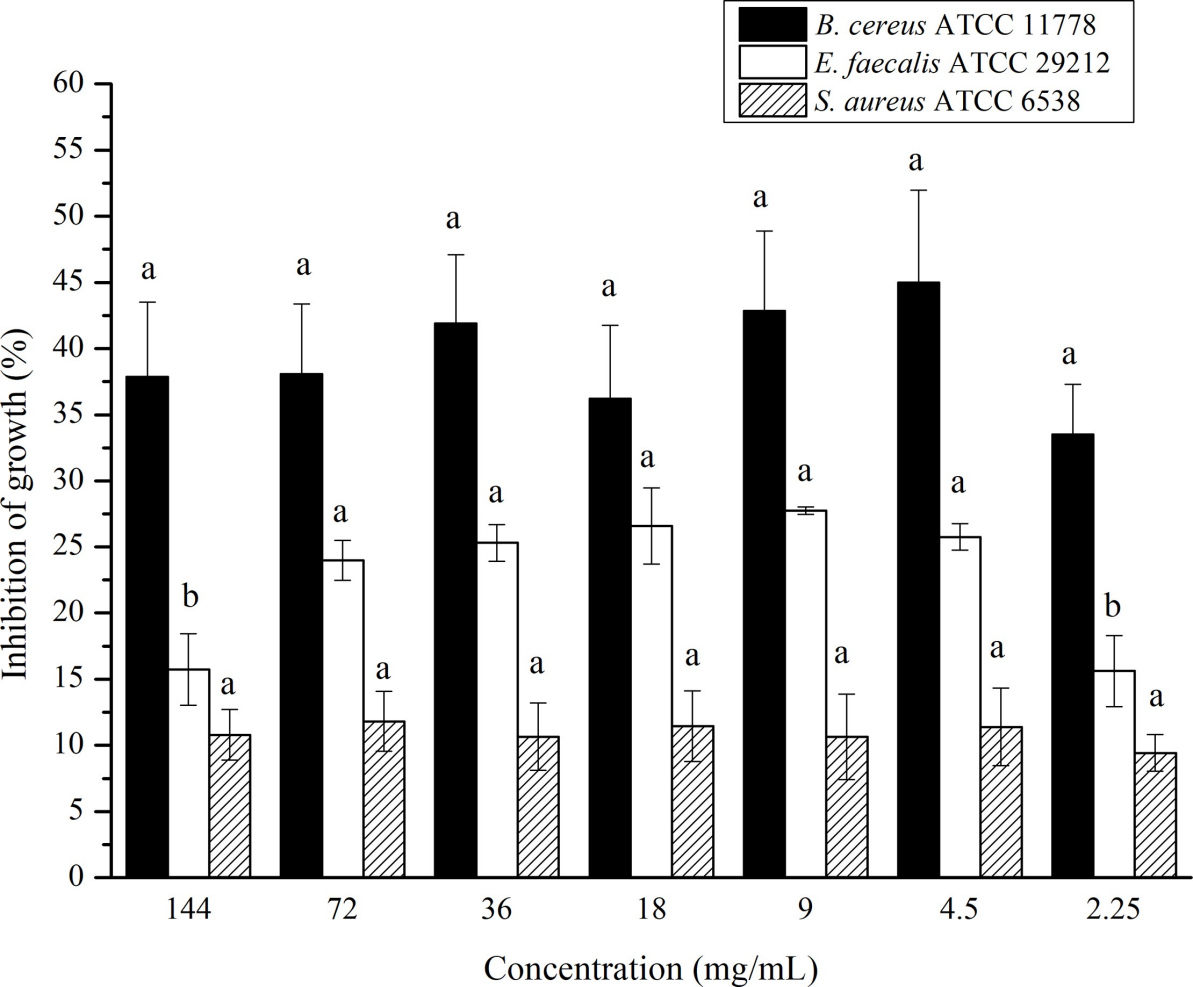
Antibacterial activity of the cacay oil in relation to Gram-positive bacteria. Different letters on the same strain indicate that there is a statistical difference between the means at 95% confidence by the Tukey test (p<0.05).

Natural oils have high biological potential due to the broad diversity of bioactive components [10]. Thus, the antibacterial activity *in vitro* of cacay oil was evaluated due to the higher concentration of polyunsaturated fatty acids (58.3%), since the antibacterial activity can be attributed to the presence of these compounds [39, 40]. Fig 2 shows the inhibition of bacterial growth of cacay oil in relation to Gram-positive bacteria.

Cacay oil showed active biological properties to inhibit the bacterial growth for Gram-positive *B*. *cereus*, *E*. *faecalis*, and *S*. *aureus* strains at all tested concentrations. However, there was a decrease in the inhibition of bacterial growth in the concentrations of 144.00 and 2.25 mg/mL for *E*. *faecalis*. It is possible that, like some antimicrobial agents, the cacay oil had no exerted antibacterial action in a concentration-dependent manner. Physicochemical factors, such as solubility and diffusion, may account for this [41].

On the other hand, no inhibition was observed for Gram-negative *E*. *coli*, *P*. *aeruginosa*, and *S*. *paratyphi* strains. This was probably due to structural differences in the outer membrane of bacteria. The thick layer of the lipopolysaccharide outer membrane of Gram-negative bacteria may have shown to be more resistant to a hydrophobic substance (cacay oil) compared with the Gram-positive bacteria, which possess a single peptidoglycan layer structure [42]. According to Meng et al. [43], the antibacterial effect of vegetable oils is due to the chemical composition which can provide a synergistic effect and affect bacterial integrity, penetrating through the cell wall and inhibiting the cellular respiration process.

To the best of our knowledge, this is the first report regarding the antibacterial effects of cacay oil. Thus, given this potential of cacay oil, the results of the present study may support further research aimed at applying oil in topical formulations for skincare and repair. In addition, the use of cacay oil can be a useful strategy for obtaining products with a longer shelf life, as well as safer products due to their ability to slow or prevent the growth of contaminating bacteria.

## Conclusions

Oils and butter evaluated in the present study had distinct composition profiles and physicochemical characterization. Coconut oil and cacay butter showed high potential to oxidative stability due to high contents of saturated fatty acids such as lauric, myristic, and palmitic acids, which influenced the iodine value. Regarding cacay oil, its high concentration of polyunsaturated fatty acids (PUFA) such as linoleic acid and being a rich source of phenolic compounds presents it as an alternative source of raw materials for the cosmetic and biotechnology industries, which can be used as health promoting products. Satisfactory results in antioxidant and antibacterial activity reinforce its commercial exploitation. Furthermore, it is expected that additional refining of the oils will improve the product characteristics and stability.

## Supporting information

S1 Dataset (XLSX)
